# Exposure to COVID-19 during the First and the Second Wave of the Pandemic and Coronavirus-Related PTSD Risk among University Students from Six Countries—A Repeated Cross-Sectional Study

**DOI:** 10.3390/jcm10235564

**Published:** 2021-11-26

**Authors:** Dominika Ochnik, Aleksandra M. Rogowska, Cezary Kuśnierz, Monika Jakubiak, Magdalena Wierzbik-Strońska, Astrid Schütz, Marco J. Held, Ana Arzenšek, Iuliia Pavlova, Elena V. Korchagina, Imran Aslan, Orhan Çınar

**Affiliations:** 1 Faculty of Medicine, University of Technology, 40-555 Katowice, Poland; rektorat@wst.com.pl; 2 Institute of Psychology, University of Opole, 45-052 Opole, Poland; arogowska@uni.opole.pl; 3 Faculty of Physical Education and Physiotherapy, Opole University of Technology, 45-758 Opole, Poland; c.kusnierz@po.edu.pl; 4 Faculty of Economics, Maria Curie-Sklodowska University in Lublin, 20-031 Lublin, Poland; monika.jakubiak@poczta.umcs.lublin.pl; 5 Department of Psychology, University of Bamberg, 96047 Bamberg, Germany; astrid.schuetz@uni-bamberg.de (A.S.); marco.held@uni-bamberg.de (M.J.H.); 6 Faculty of Management, University of Primorska, 6101 Koper, Slovenia; ana.arzensek@fm-kp.si; 7 Department of Theory and Methods of Physical Culture, Lviv State University of Physical Culture, 79007 Lviv, Ukraine; pavlova.j.o@gmail.com; 8 St. Petersburg School of Economics and Management, HSE University, 194100 St. Petersburg, Russia; elena.korchagina@mail.ru; 9 Institute of Industrial Management, Economics and Trade, Peter the Great St. Petersburg Polytechnic University, 195251 St. Petersburg, Russia; 10 Health Management Department, Bingöl University, 12000 Bingöl, Turkey; imranaslan@gmail.com; 11 Faculty of Economics and Administrative Sciences, Ataturk University, 25240 Erzurum, Turkey; orhanar@gmail.com

**Keywords:** COVID-19, exposure to COVID-19, PTSD, students, cross-national

## Abstract

This study aimed to reveal differences in exposure to coronavirus disease (COVID-19) during the first (W1) and the second (W2) waves of the pandemic in six countries among university students and to show the prevalence and associations between exposure to COVID-19 and coronavirus-related post-traumatic stress syndrome (PTSD) risk during W2. The repeated cross-sectional study was conducted among university students from Germany, Poland, Russia, Slovenia, Turkey, and Ukraine (W1: n = 1684; W2: n = 1741). Eight items measured exposure to COVID-19 (regarding COVID-19 symptoms, testing, hospitalizing quarantine, infected relatives, death of relatives, job loss, and worsening economic status due to the COVID-19 pandemic). Coronavirus-related PTSD risk was evaluated by PCL-S. The exposure to COVID-19 symptoms was higher during W2 than W1 among students from all countries, except Germany, where, in contrast, the increase in testing was the strongest. Students from Poland, Turkey, and the total sample were more frequently hospitalized for COVID-19 in W2. In these countries, and Ukraine, students were more often in quarantine. In all countries, participants were more exposed to infected friends/relatives and the loss of a family member due to COVID-19 in W2 than W1. The increase in job loss due to COVID-19 was only noted in Ukraine. Economic status during W2 only worsened in Poland and improved in Russia. This was due to the significant wave of restrictions in Russia and more stringent restrictions in Poland. The prevalence of coronavirus-related PTSD risk at three cutoff scores (25, 44, and 50) was 78.20%, 32.70%, and 23.10%, respectively. The prediction models for different severity of PTSD risk differed. Female gender, a prior diagnosis of depression, a loss of friends/relatives, job loss, and worsening economic status due to the COVID-19 were positively associated with high and very high coronavirus-related PTSD risk, while female gender, a prior PTSD diagnosis, experiencing COVID-19 symptoms, testing for COVID-19, having infected friends/relatives and worsening economic status were associated with moderate risk.

## 1. Introduction

The novel coronavirus disease 2019 (COVID-19) caused by the SARS-CoV-2 virus has become a highly viral and infectious disease globally. The World Health Organization (WHO) [1] declared the COVID-19 pandemic on 11 March 2020. The pandemic is an unexpected, global phenomenon that has affected people not only by direct exposure to the disease but also indirectly via its various consequences, e.g., economic. The COVID-19 pandemic is the most profound global economic recession in the last eight decades [2]. Additionally, research shows that mental health problems associated with the pandemic extend to the general population and are not exclusively limited to individuals who have been infected [3]. Therefore, due to financial instability, the current pandemic can affect the mental health of individuals who are not at severe risk of becoming infected with COVID-19. The COVID-19 pandemic has considerably affected mental health. The review of mental health epidemiology indicates that a psychiatric epidemic cooccurs with the COVID-19 pandemic [4].

One group that is particularly susceptible to mental health deterioration during the ongoing pandemic is university students. Research has shown that student status (being a student) predicts mental health deterioration risk [5,6,7,8]. Thus, the education sector has been strongly disturbed by the COVID-19 pandemic [9]. The factors contributing to students’ mental health issues in the pre-pandemic period are academic pressure [10] and financial obligations that may lead to poorer performance [11], and health concerns [12]. The additional risk factor of mental health problems is a young age. Even though young adults are less susceptible to COVID-19 infection [13], they are more susceptible to mental health issues during the ongoing pandemic [14,15,16].

### Post-Traumatic Stress Disorder (PTSD) and the COVID-19 Pandemic

Post-traumatic stress disorder (PTSD) is in the category of trauma- and stressor-related stress disorders [17]. The DSM-4 criteria for PTSD relating to exposure assumed that the person experienced or was confronted with an event involving actual or threatened death or serious injury or a threat to the physical integrity of one’s self or others (A1) and second, that the person’s response involved intense fear, helplessness, or horror (A2) [17]. However, in the DSM-5, significant changes have been introduced. The DSM-5 requires certain triggers, whether directly experienced, witnessed, or happening to a close family member or friend, but exposure through media is excluded unless the exposure is work-related. In addition, the second criterion of subjective response (A2) has been removed [18].

Pandemics are classified as natural disasters. PTSD is one of the most-studied psychiatric disorders and is related to natural disasters [19]. However, the DSM-5 definition notes that a life-threatening illness or debilitating medical condition is not necessarily a traumatic event. Therefore, there is a claim that exposure to the COVID-19 pandemic cannot be treated as a traumatic experience causing PTSD due to the new criteria in the DSM-5 [20]. There is an ongoing debate regarding the possibility of the anticipatory threat of the COVID-19 pandemic to be a traumatic experience and, therefore, the possibility of psychological responses coherent with PTSD [21]. Additionally, recent research [22] strongly supports this claim and emerging research in this area. Following that research, we recognize the COVID-19 pandemic as a traumatic stressor event that can cause a PTSD-like response.

Probable PTSD related to the pandemic ranges from 7% to even 67% in the general population [20]. A meta-analysis of 14 studies conducted during the first wave of the pandemic, between February and April, revealed a high rate of PTSD (23.88%) in the general population [23]. The prevalence rate of PTSD in students presents a wide range of variety. In the group of home-quarantined Chinese university students (*n* = 2485) one month after the breakout, the prevalence was 2.7%. However, Chi et al. [24] revealed that in a sample of Chinese students (*n* = 2038), the prevalence of clinically relevant PTSD reached 30.8% during the pandemic. Among a large sample of French university students (*n* = 22883), the rate of probable PTSD one month after the COVID-19 lockdown was 19.5% [25].

The predictors of PTSD in the Chinese university student sample were older age, knowing people who had been isolated, higher level of anxious attachment, adverse experiences in childhood, and lower level of resilience. However, gender, family intactness, subjective socioeconomic status (SES), and the number of confirmed cases of COVID-19 in participants’ areas turned out to be irrelevant predictors [24]. Previous research showed that typically, women show higher rates of PTSD than men [26]. PTSD usually occurs almost twice as much in women compared to men [27]. This was also proven after natural disasters (earthquakes) among young adults [28]. However, gender role in PTSD prevalence was not confirmed during the COVID-19 pandemic. The meta-analysis showed that gender was not a significant moderator of PTSD [23]. Additionally, there is strong evidence that prior mental health disorders, particularly anxiety and depression, are predictors of PTSD [29]. Furthermore, previous exposure to traumatic events is a risk factor for PTSD [30].

The research showed a significant association between exposure to COVID-19 and the severity of PTSD symptoms in university student samples [25,31]. General exposure to COVID-19 turned out to be a significant risk factor for anxiety in Czech, Polish, Turkish, and Ukrainian university students while irrelevant for anxiety in Colombian, German, Israeli, Russian, and Slovenian students during the first wave of the pandemic [32]. The same study showed that also depression risk is associated with general exposure to COVID-19 among university students from the Czech Republic, Israel, Russia, Slovenia, and Ukraine. However, in Colombia, Germany, Poland, and Turkey, the exposure was irrelevant to depression risk among university students [32].

In the present study, we will refer to university students from six countries: Germany, Poland, Russia, Slovenia, Turkey, and Ukraine between the first wave (May–June 2020) (W1) and the second wave (mid-October–December 2020) (W2) of the COVID-19 pandemic. The countries in our study represent the cultural diversity depicted by traditional vs. secular and survival vs. self-expression values. The Inglehart—Welzel World Cultural Map [33] aggregates all countries into eight clusters based on the dimensions of those values. Four out of eight value clusters are exemplified in our study. Protestant Europe is represented by Germany; Catholic Europe by Poland and Slovenia; Orthodox Europe by Ukraine and Russia; and the African-Islamic region by Turkey. Therefore, these countries represent a great diversity of global cultural values.

**Figure 1:**
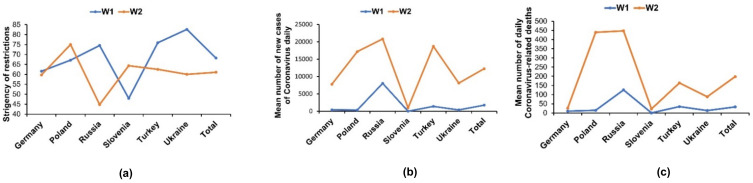
Figures present the following data in six countries (Germany, Poland, Russia, Slovenia, Ukraine, and Turkey) during the first (W1) and the second (W2) wave of the COVID-19 pandemic: (**a**) stringency of restrictions; (**b**) mean number of new daily cases of COVID-19; (**c**) mean number of new daily COVID-19-related deaths.

To present the ongoing pandemic situation in each of the six countries, we refer to the Oxford COVID-19 Government Response Tracker (OxCGRT), which enables tracking the stringency of government responses to the COVID-19 pandemic across countries and time [34]. The mean stringency index value varied in the W1 varied between 47.91 in Slovenia and 82.64 in Ukraine. During the W2, the lowest index was observed in Russia (44.80), while the highest was in Poland (75.00). The greatest increase of the OxCGRT was noted in Slovenia, while the greatest decrease of the index was in Ukraine. The detailed description of the stringency of restrictions in six countries during W1 and W2 is shown in Figure 1a. Since the national restrictions mainly refer to closing workplaces and economic measures, we assumed that in the countries that significantly waved the restrictions during W2 (e.g., Russia), the portion of university students who reported exposure to the COVID-19 pandemic in terms of losing a job and deterioration of the economic status would be lower during W2. We also analyzed the mean number of daily new cases and deaths based on an interactive web-based dashboard to track COVID-19 [35] (mean of the first and the last day of conducting the study in each country during the first and the second wave). The data on the mean number of daily cases presented in Figure 1b and on the mean number of deaths in Figure 1c show that in four countries (Germany, Russia, Turkey, and Ukraine), despite the higher number of daily cases and deaths due to COVID-19 during W2, the restrictions decreased. The largest increase in daily cases and deaths during W2 compared to W1 was noted in Poland, Russia, Turkey, and Ukraine. Our following hypothesis was that in countries with a higher number of cases and deaths during W2, the proportion of students reporting higher exposure to COVID-19 (symptoms, testing, hospitalizing, being in a strict 14-day quarantine, having infected friends/family, and experiencing death of friends/relatives) in W2 would be higher compared to W1.

The main aim of this study was to verify the differences in the exposure to the COVID-19 pandemic in university students from Germany, Poland, Russia, Slovenia, Turkey, and Ukraine between the first wave (W1) and the second wave (W2) of the COVID-19 pandemic. We expected significant differences in various aspects of exposure to COVID-19 dependent on country, which might be interpreted in the context of stringency of restrictions and the number of daily cases and deaths due to the coronavirus. 

In this study, we acknowledge the COVID-19 pandemic as a traumatic stressor event that can cause a PTSD-like response. The second aim is to reveal whether different aspects of exposure to COVID-19 (symptoms, testing, hospitalizing, being in quarantine, having infected friends/family, experiencing the death of friends/relatives, losing a job, worsening of economic status), including previous diagnosed mental health problems (depression, anxiety, PTSD) and gender predict coronavirus-related PTSD severity risk in international samples of university students from six countries during W2.

This study fills the gap in the literature related to the link between exposure to the COVID-19 pandemic and coronavirus-related PTSD during the second wave of the pandemic among students from six countries.

## 2. Materials and Methods

### 2.1. Participants 

The required sample size for each country group was computed a priori using the G*Power software (Düsseldorf, Germany) [36]. To detect a medium effect size of Cohen’s W = 0.03 with 95% power in a 2 × 2 χ^2^ contingency table, df = 1 (two groups in two categories each, two-tailed), α = 0.05, G*Power suggests we would need 145 participants in each country group (non-centrality parameter λ = 13.05; critical χ^2^ = 3.84; power = 0.95). All the respondents were eligible for the study and confirmed their student status (being a current university student).

The cross-sectional study was conducted in six countries with a total of 1684 students during the first wave of the pandemic—in Germany (*n* = 270, 16%), Poland (*n* = 300, 18%), Russia (*n* = 285, 17%), Slovenia (*n* = 209, 13%), Turkey (*n* = 310, 18%), and Ukraine (*n* = 310, 18%)—and a total of 1741 during the second wave, in Germany (*n* = 276, 16%), Poland (*n* = 341, 20%), Russia (*n* = 274, 15%), Slovenia (*n* = 206, 12%), Turkey (*n* = 312, 18%), and Ukraine (*n* = 332, 19%).

The total sample of German students was recruited from University of Bamberg during the first measurement (W1) (*n* = 270, 100%) and the second measurement (W2) (*n* = 276, 100%). The Polish sample during W1 consisted of 300 students recruited from Maria Curie-Sklodowska University (UMCS) in eastern Poland (*n* = 149, 49%) and from University of Opole (UO) in the south of Poland (*n* = 151, 51%). During W2, Polish sample was comprised of 341 students from the same universities: UMCS (*n* = 57, 17%) and UO (*n* = 284, 83%). There were 285 Russian students in W1 and 274 in W2. Russian students were recruited from universities located in Sankt Petersburg: Peter the Great St. Petersburg Polytechnic University (W1: *n* = 155, 54%; W2: *n* = 156, 54%), Higher School of Economics (HSE) University (W1: *n* = 90, 31%; W2: *n* = 39, 14%), and St. Petersburg State University of Economics and Finance (W1: *n* = 42, 15%; W2: *n* = 78, 29%). The total sample in Slovenia was comprised of students recruited from University of Primorska in Koper during W1 (*n* = 209, 100%) and W2 (*n* = 206, 100%). During W1, Turkish students were recruited from eleven Turkish universities, mostly located in eastern Turkey: Bingol University, Bingöl (*n* = 148, 48%); Atatürk University, Erzurum (*n* = 110, 35%); Muğla Sıtkı Koçman University, Muğla (*n* = 35, 11%); Ağrı İbrahim Çeçen University, Ağrı (*n* = 6, 2%); Fırat University, Elazığ (*n* = 3, 0.8%); Kırıkkale University, Kırıkkale (*n* = 1, 0.3%); Adnan Menderes University, Aydın (*n* = 1, 0.3%); Başkent University, (*n* = 3, 1%); Boğaziçi University (*n* = 1, 0.3%), Dicle University, Diyarbakır (*n* = 1, 0.3%), and Istanbul University (*n* = 1, 0.3%). During W2, Turkish students were recruited from seven Turkish universities: Atatürk University, Erzurum (*n* = 110, 35%); Ağrı İbrahim Çeçen University, Ağrı (*n* = 71, 23%); Bingol University, Bingöl (*n* = 57, 18%); Iğdır University, Iğdır (*n* = 26, 8%); Muğla Sıtkı Koçman University, Muğla (*n* = 20, 7%); Başkent University, (*n* = 16, 5%); and Bursa Uludağ University, Bursa (*n* = 12, 4%). Ukrainian students represented Lviv State University of Physical Culture (W1: *n* = 310, 100%; W2: *n* = 332, 100%;).

Female students constituted 70% of the sample (*n* = 1174) during W1 and 73% (*n* = 1275) during W2. The majority of the participants lived in rural areas and small towns in W1 (*n* = 1021, 61%) and in W2 (*n* = 1029, 59%). Most of students were at the first cycle studies (bachelors’ level) (W1: *n* = 1269, 75%; W2: *n* = 1324, 76%). The average age was 22.80 (SD = 4.65) in W1 and 22.73 (SD = 3.86) in W2. The median of age was 22.

Students reported prior professional diagnosis of depression (*n* = 356, 20.40%), anxiety (*n* = 287, 16.50%), and PTSD (*n* = 205, 11.80%). The data regarding previous diagnosis in Germany were not collected due to an electronic problem.

The sociodemographic profiles of the participants in W1 and W2 are highly similar and comparable. Detailed descriptive statistics and previous diagnoses of depression, anxiety, and PTSD for each country during W1 and W2 are presented in Table 1.

All the questions included in the Google Forms questionnaire were answered in Poland, Slovenia, Czechia, Ukraine, and Russia. In those countries, participants could not omit any response; therefore, there were no missing data. However, in the German sample, the study was conducted via SoSci Survey, and there were missing data (*n* = 5, 0.02%). Therefore, hot-deck imputation was introduced to deal with a low number of missing data in the German sample.

### 2.2. Study Design

This repeated cross-sectional study among students from Germany, Poland, Russia, Slovenia, Turkey, and Ukraine was conducted during the first wave (W1) (May–June 2020) and the second wave (W2) (mid-October–December 2020) of the pandemic. The first measurement (W1) results concerning depression and anxiety have been already carefully described in a previous publication [32].

A cross-national first measurement was conducted online between May and June in the following countries: Germany (2–25 June), Poland (19 May–25 June), Russia (01–22 June), Slovenia (14 May–26 June), Turkey (16–29 May), and Ukraine (14 May–02 June). The second measurement during W2 was conducted between mid-October and December 2020 in Germany (15 October–1 November), Poland (11 November–1 December), Russia (28 October–8 December), Slovenia (10 October–15 December), Turkey (18 November–8 December), and Ukraine (15 October–15 November).

The survey study was conducted via Google Forms in all countries except Germany. This country exploited the SoSci Survey. The invitation to participate in the survey was sent to students by researchers via various means, e.g., Moodle e-learning platform, student offices, email, or social media. The average time of data collection was 23.26 min (SD = 44.03). In Germany, students were offered a possibility to enter the lottery for a 20 EUR Amazon gift card in W1 and 50 EUR in W2 as an incentive to participate. No form of compensation was offered as an incentive to participate in the five other countries. To minimize bias sources, the student sample was highly diversified regarding its key characteristics: the type of university, field of study, and the cycle of study. Sampling was purposive. The selection criterion was university student status. The study followed the ethical requirements of the anonymity and voluntariness of participation.

### 2.3. Measurements

#### 2.3.1. Sociodemographic Survey

Demographic data included questions regarding gender, place of residence (village, town, city, agglomeration), the current level of study (bachelor, master, postgraduate, doctoral), field of study (social sciences, humanities, and art, natural sciences, medical and health sciences), the year of study, and the study mode (full-time vs. part-time). The questionnaire was primarily designed in Polish and English. In the second step, it was translated from English to German, Russian, Slovenian, Turkish, and Ukrainian using backward translation by a team consisting of native speakers and psychology experts according to guidelines [37]. The participants were asked about their previous medical conditions regarding depression, anxiety, and PTSD diagnosed by a doctor or other licensed medical provider. The answer ‘yes’ was coded as 1, ‘no’ as 0.

#### 2.3.2. Self-Reported Exposure to COVID-19

Exposure to COVID-19 [38] was assessed based on eight questions regarding the COVID-19 pandemic in terms of (1) symptoms that could indicate coronavirus infection; (2) being tested for COVID-19; (3) hospitalization due to COVID-19; (4) experiencing strict quarantine for at least 14 days, in isolation from loved ones due to COVID-19; (5) coronavirus infection among family, friends, or relatives; (6) death among relatives due to COVID-19; (7) losing a job due to the COVID-19 pandemic—the person or their family; and (8) experiencing a worsening of economic status due to the COVID-19 pandemic. Participants marked their answers to each question, coded as 0 = no, and 1 = yes. Each aspect of the exposure to COVID-19 was analyzed separately. The self-exposure to COVID-19 items was developed based on methodology proposed by Tang et al. [31].

#### 2.3.3. Coronavirus-Related PTSD

The coronavirus-related PTSD was assessed using the 17-item PTSD check list—specific version (PCL-S) [39] on a five-point Likert scale ranging from 1 = not at all to 5 = extremely, with the total score ranging from 17 to 85. Higher scores indicated higher PTSD levels. A lower cutoff score (25) [40] is used for screening reasons. However, higher cutoff points (44) and (50) [41] are dedicated to minimalizing false positives or diagnoses.

We have used PCL-S based on the DSM-4, as we wanted to be sure that we measure coronavirus-related PTSD. The specific stressful-event-related PTSD was acknowledged as the COVID-19 pandemic. Therefore, we have utilized the specific version and asked about symptoms in response to a specific stressful experience: the COVID-19 pandemic. We have also added the COVID-19 pandemic aspect to each of the items. Participants estimated how much they were bothered by this specific problem (the COVID-19 pandemic) in the past month. Therefore, we have not explored general PTSD but specific stressful-event-related PTSD. The Cronbach’s α in the total sample in this study was 0.94.

#### 2.3.4. Stringency Index

We used the Oxford COVID-19 Government Response Tracker (OxCGRT) to portray the stringency of government responses to the COVID-19 pandemic across countries and time [34]. The stringency level is composed of various indicators. It refers to community mobility: restrictions on gathering, workplace closings, public school closings, cancelation of public events, stay at home requirements, transport closings, international travel restrictions, restrictions on internal movement, and economic measures: fiscal measures, income support, debt/contract relief, and international support. The indices regarding public health issues are: testing policy, public information campaigns, contact tracking, investments in vaccines, emergency investment in health care, vaccination, and facial coverings. The stringency of government responses is the reaction to the pandemic spread in each country. Those measurements are rescaled to a value ranging from 0 to 100, where 100 denotes the strictest restrictions. The timing was crucial for the stringency-level evaluation. The stringency rate in this study was calculated based on the stringency value mean in the first and the last day of data collection in each country. This index portrays the pandemic situation for the general population in each country well.

### 2.4. Statistical Analysis

The statistical analysis included descriptive statistics: mean (M), standard deviation (SD), and 95% of confidence interval (CI) with lower limit (LL) and upper limit (UL). The analysis was conducted in SPSS27. To verify the first hypothesis regarding the change in exposure to COVID-19, we have utilized the Pearson χ^2^ independence test for each country and each aspect of exposure to COVID-19 separately using a 2 × 2 contingency table. Phi (φ) value was used to assess the effect size [42]. An effect size equal to 0.1 is considered a small effect, 0.3 a medium effect, and 0.5 a large effect. We have shown the prevalence rate for coronavirus-related PTSD. The following step was to verify whether the various aspects of the COVID-19 pandemic exposure are associated with coronavirus-related PTSD in university students. We conducted multivariate logistic regression analysis for the coronavirus-related PTSD risk among the international student sample from the six countries. All predictors were entered into the model simultaneously. The multiple regression models reveal risk factors in their simultaneous effect on mental health. Therefore, the multivariate regression model is closer to actual psychological complexity than the bivariate model, where the particular factors independently predict mental health issues.

## 3. Results

The Person’s χ^2^ independence test showed a significant difference between measurement during W1 (May–June 2021) and W2 (mid-October–November) in each of the six countries regarding the various aspects of self-reported exposure to COVID-19. The ϕ coefficient value allowed for the assessment of the effect size [42].

### 3.1. Comparison of Self-Reported Exposure to the COVID-19 Pandemic

A significantly higher proportion of students experienced symptoms of coronavirus infection during the second wave in the total international sample of university students. However, the effect size was small. Similarly, in Poland, Russia, Slovenia, and Turkey, the proportion of students experiencing COVID-19 symptoms was significantly higher in W2, although the effect size was small. A significant medium effect size was noted in Ukraine. Therefore, the most pronounced increase in the proportion of students experiencing the COVID-19 symptoms during the second wave was observed in Ukraine. However, the one country where there was no significant effect was Germany. Therefore, the university students in Germany did not experience higher exposure to the infection in the second wave, unlike all other students from the five countries.

However, a significant medium effect sized was observed in German students regarding testing for coronavirus. In all other countries and the total sample, the effect was also significant but small. Therefore, all university students reported a higher number of tests in W2, but the difference was the highest in Germany.

The exposure to being hospitalized for coronavirus was relatively small. Only five participants (0.30%) in W1 and 21 (1.21%) answered yes to this question in the total sample. However, the difference was significant. A significantly higher proportion of students was hospitalized in Poland and Turkey during W2, although the effect size was small. In Germany, Russia, Slovenia, and Ukraine, the difference was insignificant.

A higher proportion of students experienced being in a strict quarantine during W2 than W1 in Poland, Turkey, Ukraine, and the total sample. However, in Germany, Russia, and Slovenia, the differences were trivial.

In all countries and the total international sample, the exposure to friends or relatives infected with the COVID-19 was higher during W2 than W1. A large significant effect was observed in Turkey, a medium effect in Ukraine and the total sample, while a small effect was observed in Germany, Poland, Russia, and Slovenia.

Similarly, the proportion of students who experienced a loss of friends or relatives due to the COVID-19 significantly increased during W2 compared to W1. The medium effect was observed in Turkey, while a small effect was prevalent in all other countries and the international sample.

The proportion of students who experienced losing a job due to the COVID-19 pandemic was lower during W2 than W1 in the international sample and Ukraine. However, in other countries, the effect size was small. There was no significant drop in Germany, Poland, Russia, and Turkey.

Mixed results were observed regarding the self-reported deterioration of economic status due to the pandemic. In the total sample, the difference between W1 and W2 was trivial. However, an increase in the proportion of students declaring that their economic status worsened was observed in Poland. On the other hand, there was a significant drop in the proportion of students claiming worse economic status during W2 in Russia. All effects were small regarding this aspect of exposure. There were no significant differences in Germany, Slovenia, Turkey, and Ukraine. The results of the comparison are shown in Table 2.

### 3.2. Descriptive Statistics and Prevalence of Coronavirus-Related PTSD

Descriptive statistics showed that the mean value of coronavirus-related PTSD was 38.08 (SD = 15.49) among students from Germany, Poland, Russia, Slovenia, Turkey, and Ukraine during W2. A detailed description is presented in Table 3.

The prevalence of coronavirus-related PTSD risk was presented at three cutoff points, according to the recommendations in the presented literature [40,41]. The proportion of students with coronavirus-related PTSD risk at three cutoff scores (25, 44, and 50) is presented in Table 4.

### 3.3. Logistic Regression for Coronavirus-Related PTSD Risk

Multivariate logistic regression for coronavirus-related PTSD risk during the second pandemic wave showed significant models for a moderate, high, and very high risk of PTSD among an international sample of university students from Germany, Poland, Russia, Slovenia, Turkey, and Ukraine. The predictors were eight aspects of self-reported exposure to COVID-19 controlling for gender and previous clinical diagnosis of depression, anxiety disorder, and PTSD. All predictors were included simultaneously using the enter method. Results are presented in Table 5.

The model of moderate risk of coronavirus-related PTSD (Cutoff Point 25) revealed only three predictors to be relevant among eight items describing exposure to the coronavirus pandemic: experiencing COVID-19 symptoms (Item 1), COVID-19 infection among friends and family (Item 5), and the deterioration of economic status due to the pandemic (Item 8). Students who experienced COVID-19 symptoms and whose family or friends were infected had 1.5 times higher odds of moderate risk of PTSD. Those who reported worsening economic status due to the pandemic were almost two and half times more frequently in the moderate PTSD risk group. In addition, female students were two times more likely to develop moderate PTSD. Coronavirus-related PTSD was three times more likely among students with a previous clinical diagnosis of PTSD.

The regression models for high and very high risk of PTSD revealed a different set of predictors. In those two models, the significant predictors were the same with similar adjusted odds. Students who had a family member or friend die from coronavirus infection were twice as likely to be in a coronavirus-related PTSD-risk group. Additionally, students exposed to the COVID-19 pandemic in terms of losing a job (own or in the one’s family) and worsening economic status were 1.6 times and 1.8 times more likely to be in a (very) high coronavirus-related PTSD-risk group, respectively. Finally, worsening of economic status was a significant predictor of high and very high risk of PTSD. Among demographic factors, female gender and previous diagnosis of depression and PTSD were associated with a twofold higher risk of coronavirus-related PTSD.

## 4. Discussion

In this study, we showed the significance of differences in aspects of exposure to the COVID-19 pandemic in university students from Germany, Poland, Russia, Slovenia, Turkey, and Ukraine between the first wave (W1) and the second wave (W2) of the COVID-19 pandemic with regard to the stringency index. We also showed the prevalence and predictors of coronavirus-related PTSD. To the authors’ knowledge, this is the first study undertaking this theme among university students from eight countries during W2.

Our study revealed the differences in exposure to COVID-19 among university students in Germany, Poland, Russia, Slovenia, Ukraine, and Turkey during W1 (April–May 2020) and W2 (October–December 2020). The prevalence of coronavirus-related PTSD risk for 25, 44, and 50 cutoff scores was 78.20%, 32.70%, and 23.10%, respectively, during W2. We have also performed the prediction models of coronavirus-related PTSD risk for each cutoff score in the international sample of university students during W2.

We expected that in countries such as Russia, where the restrictions were significantly waved during W2, the worsening of economic status and job loss due to the COVID-19 pandemic would significantly decrease. The mean stringency of restrictions in the six countries was lower during W2 compared to W1. However, the ratio of students in the international sample who have lost a job during W2 was significantly lower compared to W1. In contrast, the ratio of students whose economic status worsened due to the pandemic was not significantly different during W2. Therefore, the most significant job loss experience by a student or a family member was more evident during W1 (31%) than W2 (25%). However, the deterioration of economic status was still on the rise even during W2 (although insignificant) and concerned over half of the international student sample (55%). The lowest proportion of students exposed to worsening economic during W2 was noted in Germany (29.92%), while the highest (over 50%) in Poland, Ukraine, and Turkey, at 72.14%, 70.41%, and 63.78%, respectively. In contrast, the proportion of French students who reported a loss of income was significantly lower and reached only 18.30% in June–July 2020 [25]. In accordance with our expectations, the rate of students who experience worsening economic status due to the pandemic was significantly lower in Russia during W2 due to the significant wave of the restrictions, whereas it was higher in Poland, where the restrictions were more stringent.

In congruence with Hypothesis 2, exposure to COVID-19 among the total sample of students has risen. During W2, a higher proportion of students in all countries reported experiencing symptoms of COVID-19 compared to W1, except Germany, even though the number of new cases daily was almost 20 times higher during W2 (*n* = 7762) than during W1 (*n* = 392) in the general German population. On the other hand, the difference in the frequency of testing for COVID-19 was the largest in the German sample. Therefore, although the ratio of German students who experienced having infected friends/family or losing a loved one was higher during W2, the portion of German students who experienced COVID-19 has not increased. It might be due to the significant increase in testing among German students.

There was significant growth in the percentage of hospitalized students in strict quarantine in Poland and Turkey. Additionally, in Ukraine, the ratio of students in a compulsory 14-day quarantine was elevated during W2. In congruence with the numbers in the general population, the percentage of students who experience losing a family member or friends due to COVID-19 was higher in all countries. However, the largest increase of daily coronavirus-related deaths was among the Polish and Russian general populations. In contrast, among the student population, the highest increase was declared in Turkey. Similar to previous research among Turkish students [43], it would seem that the student sample was overexposed to the bereavement experience. However, there were concerns regarding the reliability of COVID-19 data in Turkey, as it appeared that the prevalence of the disease (particularly total deaths) might be underreported [44,45].

The mean for the coronavirus-related PTSD risk in the international sample of students from six countries in this study has exceeded the lowest cutoff score (25), which is used for screening reasons [40]. The prevalence at this cutoff point was very high and indicated that over 78.20% of students are at coronavirus-related PTSD risk in this study. Every third student (32.70%) is at high PTSD risk (Cutoff Point 44), and almost every fourth student (23.10%) is at a very high PTSD risk (Cutoff Point 50). The high cutoffs are used to minimalize false positives or diagnoses [41]. The prevalence of PTSD risk at the beginning of the first wave of the COVID-19 pandemic in young adults in the USA [46] and China [16] with the use of PCL-C was 32% (Cutoff Point 44) and 14% (Cutoff Point 38), respectively. Research with the use of the PCL-5 at Cutoff Point 32 in the general population showed a total of 7% of people experiencing post-traumatic stress symptoms in the Chinese sample (January/February, cutoff score—33) [47] and 13% in five western countries [22]. However, the Italian general sample, using a modified 19-item PCL-5-based-PTSD questionnaire, revealed a total of 29% of people experiencing PTSD symptomatology [48]. The highest prevalence (67% demonstrating high PTSD level) was in a general Chinese population, with a different measurement (IES-R) [49]. Various measurements and cutoff scores hinder the comparison to our sample. Additionally, the presented studies were conducted during the first wave of the pandemic. However, referring to the specific cutoff score (44), the prevalence of coronavirus-related PTSD risk was similar in the student sample in our study (33%) during the second wave of the pandemic among young adults in the USA (32%) [46]. On the other hand, the used PCL-C version was general and did not refer to COVID-19 as a specific stressful event [46], such as in our study. In contrast, a single-arm meta-analysis [50] of 478 papers and 12 studies showed that the prevalence of PTSD in the general population during the COVID-19 pandemic was 15%; therefore, it was significantly lower than among students in this study.

There are inconsistent data regarding the prevalence of PTSD in the student population. In French university students one month after the COVID-19 lockdown, the prevalence of PTSD risk measured by the PCL-5 (Cutoff Score 32) was 19.50% [25]. Among Chinese college students, using the abbreviated PCL, conducted in February 2020, the prevalence was 31% [24]. The smallest prevalence, reaching 2.7%, was noted in Chinese university students [31]. The measurement in this study was PCL-C, with a cutoff score of 38. The repeated cross-sectional research among French students revealed that 16.40% of students developed probable PTSD in the second measurement. The increase in the second measurement [25] can explain the high prevalence at the screening level (Cutoff Point 25) in our sample (78.20%).

The prediction models for coronavirus-related PTSD risk differed due to the severity of risk regarding the exposure to experiencing symptoms of COVID-19, testing for COVID-19, and infection of friends or family members. In the prediction model of moderate PTSD risk (Cutoff Point 25), these were important factors, while in the more severe PTSD risk models (Cutoff Points 44 and 50), they were irrelevant. The following significant predictors for the more severe PTSD risk models were experiencing symptoms of COVID-19, losing a family member or friends because of COVID-19, job loss (by the participant or family member), and worsening of economic status due to the COVID-19 pandemic. However, experiencing the loss of a friend or family member and job loss were not relevant predictors for moderate coronavirus-related PTSD risk. Testing and hospitalization for COVID-19, as well as being in strict 14-day quarantine, were not significantly associated with coronavirus-related PTSD risk in any model. The results are similar to research among Chinese students [31], where longer home quarantine was not associated with PTSD. However, in the French university sample, having lived through quarantine alone was a significant factor associated with probable PTSD [25]. The lack of association of quarantine experience with PTSD risk in this study can be due to the low proportion of exposed students (11%).

Prior medical diagnosis reported by students regarding depression was associated with high and very high coronavirus-related PTSD. Prior PTSD diagnosis was associated with a moderate and very high risk of coronavirus-related PTSD in the international sample. These results are aligned with previous findings [30]. However, prior anxiety diagnosis did not turn out to be relevant for PTSD risk in this study.

Contrary to other research [23,24] showing insignificance of gender as a PTSD moderator among young adults during the COVID-19 pandemic, we found that female students were twice as likely to develop moderate, high, or very high coronavirus-related PTSD risk. A similar assessment of PTSD risk was recognized in previous research [26,27] regarding natural disasters [28]. This inconsistency might be due to the time of the study, as the previous research shows results from the first wave of the pandemic, whereas, in our study, results come from the second wave. Due to the longer period, gender differences might be more pronounced among students.

### Limitations

There are some limitations to the present study. First, the study is of a repeated cross-sectional character and is not longitudinal. Second, the study utilized self-report questionnaires. Therefore, the results might be subject to retrospective response bias. Additionally, the research sample is convenient. The lack of representation of the student population limited to specific regions in each country seem to be a burden in generalizing the results, particularly in the Turkish case, where the majority of students come from a highly volatile region of Eastern Turkey. Additionally, we utilized the PCL-S based on the DSM-4 instead of the PCL-5 based on DSM-5. However, the PCL-S enables the measurement of PTSD with regard to a specific stressful experience: the COVID-19 pandemic. The majority of participants were female students (70%); however, this balance reflects the real gender balance in most of the surveyed countries, where the percentage of female students reaches 60% [51,52,53,54].

Considering the limitations and strengths of this study, future research directions should be the study of exposure and coronavirus-related PTSD from a cross-cultural perspective with longitudinal design in a representative sample. It should be noted that this study was conducted before introducing open public vaccination programs. We could expect that access to vaccination will mitigate the negative psychological aspect of the COVID-19 pandemic. However, students have ambivalent attitudes towards vaccination programs, particularly non-medical students [55]. Therefore, this access might also be a source of psychological distress in the future.

## 5. Conclusions

This study shows that, besides exposure to COVID-19 symptoms, the loss of relatives because of COVID-19, female gender, and a prior diagnosis of a mental health disorder, the economic aspect of the pandemic plays a vital role in the susceptibility to high coronavirus-related PTSD risk. Even though the proportion of students who have experienced worsening economic status has not increased during W2, it still considered over half of the student sample from six countries in this study. Therefore, additional financial support for students could mitigate coronavirus-related PTSD risk, particularly in Poland, Ukraine, and Turkey.

The analysis of the federal restrictions’ stringency shed light on an increase of worsening economic status in Poland (where the restrictions were more stringent) and a decrease in Russia, where the restrictions were waived despite a high number of new daily cases. The German case shows the importance of frequent testing; however, this research was conducted before open public access to the COVID-19 vaccine.

## Figures and Tables

**Table 1:** Sociodemographic characteristics and previous diagnosis of depression, anxiety, and PTSD of the study sample in the six countries.

Demographic Variables	TOTAL		Germany		Poland		Russia		Slovenia		Turkey		Ukraine	
	*n*	%	*n*	%	*n*	%	*n*	%	*n*	%	*n*	%	*n*	%
**W1**														
Gender														
Women	1174	69.70	193	71.50	220	73.40	193	67.00	178	85.20	173	55.80	217	70.00
Men	504	29.90	75	27.80	80	26.60	92	32.20	31	14.80	133	42.90	93	30.00
Did not want to say	6	0.40	2	0.70	0	0.00	0	0.00	0	0.00	4	1.30	0	0.00
Place of residence														
Village	449	26.70	53	19.60	140	46.80	7	2.50	85	40.70	52	16.80	112	36.10
Town	572	34.00	177	65.60	94	31.20	81	28.40	65	31.10	41	13.20	114	36.80
City	481	28.60	33	12.20	61	20.30	171	60.00	40	31.10	101	32.60	75	24.20
Agglomeration	182	10.70	7	2.60	5	1.70	26	9.10	19	9.10	116	37.40	9	2.90
Level of study														
Bachelor	1269	75.30	137	50.70	170	56.80	245	86.10	143	68.40	283	91.30	291	93.90
Master	340	20.20	96	36.60	130	43.20	33	11.60	61	29.20	1	0.30	19	6.10
Postgraduate	67	4.00	35	13.00	0	0.00	7	2.50	0	0.00	25	8.10	0	0.00
Doctoral	8	0.50	2	0.70	0	0.00	0	0.00	5	2.40	1	0.30	0	0.00
TOTAL	1684	100	270	100	300	100	285	100	209	100	310	100	310	100
**W2**														
Gender														
Women	1275	73.20	158	57.20	308	90.30	204	74.50	161	78.20	215	68.90	229	69.00
Men	454	26.10	116	42.00	29	8.50	70	25.50	45	21.80	97	31.10	97	29.20
Did not want to say	12	0.70	2	0.70	4	1.20	0	0.00	0	0.00	0	0.00	0	0.00
Place of residence														
Village	442	25.40	48	17.40	145	42.80	10	3.60	66	32.00	61	19.60	111	33.40
Town	587	33.70	173	62.70	140	41.10	68	24.80	80	38.80	13	4.20	113	34.00
City	559	32.10	41	14.90	50	14.70	181	66.10	52	25.20	132	42.30	103	31.00
Agglomeration	149	8.60	10	3.60	5	1.50	15	5.50	8	3.90	106	34.00	5	1.50
Other	4	0.20	4	1.40	0	0.00	0	0.00	0	0.00	0	0.00	0	0.00
Level of study														
Bachelor	1324	75.90	134	48.60	220	64.50	232	84.70	146	70.90	291	93.30	301	90.70
Master	384	22.10	122	44.20	121	35.50	38	13.90	60	29.10	12	3.80	31	9.30
Postgraduate	23	1.30	19	6.90	0	0.00	4	1.50	0	0.00	0	0.00	0	0.00
Doctoral	10	0.70	1	0.40	0	0.00	0	0.00	0	0.00	9	2.90	0	0.00
Prior diagnosis														
Depression	356	20.40	38	13.80	36	10.60	38	13.90	18	8.70	154	49.40	72	21.70
Anxiety	287	16.50	18	6.50	36	10.60	37	13.50	25	12.10	106	34.00	65	19.60
PTSD	205	11.80	-	-	8	2.30	18	6.60	3	1.50	124	39.70	52	15.70
TOTAL	1741	100	276	100	341	100	285	100	209	100	310	100	310	100

Note. W1—first wave of the COVID-19 pandemic (May–June 2020), W2—the second wave of the COVID-19 pandemic (mid-October–December 2020).

**Table 2:** Comparison of proportions of university students who experienced exposure to the coronavirus pandemic during the first wave W1 (May–June 2020) and the second wave W2 (October–December 2020) of the COVID-19 pandemic.

		Self-Reported Exposure								
Exposure Item			No		Yes			Pearson’s		
	Sample	Wave	*n*	%	*n*	%	*n*	χ^2^(1)	*p*	ϕ
1. Experiencing Symptoms of Coronavirus Infection										
	Total	W1	1486	88.24	198	11.76	1684	162.29	<0.001	0.22
		W2	1229	70.59	512	29.41	1741			
	Germany	W1	218	80.74	52	19.26	270	3.27	0.071	0.08
		W2	205	74.28	71	25.72	276			
	Poland	W1	274	91.33	26	8.67	300	22.13	<0.001	0.19
		W2	265	77.71	76	22.29	341			
	Russia	W1	237	83.16	48	16.84	285	21.66	<0.001	0.20
		W2	181	66.06	93	33.94	274			
	Slovenia	W1	181	86.60	28	13.40	209	10.75	0.001	0.16
		W2	152	73.79	54	26.21	206			
	Turkey	W1	288	92.90	22	7.10	310	53.22	<0.001	0.29
		W2	219	70.19	93	29.81	312			
	Ukraine	W1	288	92.90	22	7.10	310	84.77	<0.001	0.36
		W2	207	62.35	125	37.65	332			
2. Testing for Coronavirus										
	Total	W1	1611	95.67	73	4.33	1684	176.23	<0.001	0.23
		W2	1411	81.05	330	18.95	1741			
	Germany	W1	259	95.93	11	4.07	270	51.31	<0.001	0.31
		W2	204	73.91	72	26.09	276			
	Poland	W1	296	98.67	4	1.33	300	18.61	<0.001	0.17
		W2	310	90.91	31	9.09	341			
	Russia	W1	253	88.77	32	11.23	285	23.52	<0.001	0.21
		W2	199	72.63	75	27.37	274			
	Slovenia	W1	200	95.69	9	4.31	209	6.86	0.009	0.13
		W2	183	88.83	23	11.17	206			
	Turkey	W1	299	96.45	11	3.55	310	48.97	<0.001	0.28
		W2	242	77.56	70	22.44	312			
	Ukraine	W1	304	98.06	6	1.94	310	44.18	<0.001	0.26
		W2	273	82.23	59	17.77	332			
3. Hospitalizing for Coronavirus										
	Total	W1	1679	99.70	5	0.30	1684	9.40	<0.001	0.05
		W2	1719	98.79	21	1.21	1741			
	Germany	W1	270	100.00	0	0.00	270	1.96	0.161	0.06
		W2	274	99.28	2	0.72	276			
	Poland	W1	300	100.00	0	0.00	300	6.23	0.013	0.10
		W2	334	97.95	7	2.05	341			
	Russia	W1	283	99.30	2	0.70	285	1.92	0.166	−0.06
		W2	273	100.00	0	0.00	273			
	Slovenia	W1	209	100.00	0	0.00	209	0.00	1.00	0.00
		W2	206	100.00	0	0.00	206			
	Turkey	W1	309	99.68	1	0.32	310	4.52	0.034	0.09
		W2	305	97.76	7	2.24	312			
	Ukraine	W1	308	99.35	2	0.65	310	1.10	0.294	0.04
		W2	327	98.49	5	1.51	332			
4. Being in a Strict Quarantine for at Least 14 Days										
	Total	W1	1575	93.53	109	6.47	1684	18.82	<0.001	0.07
		W2	1556	89.37	185	10.63	1741			
	Germany	W1	246	91.11	24	8.89	270	1.46	0.227	−0.05
		W2	259	93.84	17	6.16	276			
	Poland	W1	294	98.00	6	2.00	300	9.86	0.002	0.12
		W2	316	92.67	25	7.33	341			
	Russia	W1	254	89.12	31	10.88	285	1.44	0.230	0.05
		W2	235	85.77	39	14.23	273			
	Slovenia	W1	203	97.13	6	2.87	209	1.61	0.205	0.06
		W2	195	94.66	11	5.34	206			
	Turkey	W1	293	94.52	17	5.48	310	13.84	<0.001	0.15
		W2	267	85.58	45	14.42	312			
	Ukraine	W1	285	91.94	25	8.06	310	6.50	0.011	0.10
		W2	284	85.54	48	14.46	332			
5. Friends or Relatives Were Infected										
	Total	W1	1441	85.57	243	14.43	1684	516.36	<0.001	0.39
		W2	854	49.05	887	50.95	1741			
	Germany	W1	207	76.67	63	23.33	270	4.59	0.032	0.09
		W2	189	68.48	87	31.52	276			
	Poland	W1	277	92.33	23	7.67	300	166.08	<0.001	0.51
		W2	151	44.28	190	55.72	341			
	Russia	W1	225	78.95	60	21.05	285	67.61	<0.001	0.35
		W2	124	45.26	150	54.74	273			
	Slovenia	W1	195	93.30	14	6.70	209	32.17	<0.001	0.28
		W2	149	72.33	57	27.67	206			
	Turkey	W1	242	78.06	68	21.94	310	199.20	<0.001	0.57
		W2	67	21.47	245	78.53	312			
	Ukraine	W1	295	95.16	15	4.84	310	148.84	<0.001	0.48
		W2	174	52.41	158	47.59	332			
6. Friends or Relatives Died Due to Coronavirus										
	Total	W1	1643	97.57	41	2.43	1684	131.47	<0.001	0.19
		W2	1516	87.08	225	12.92	1741			
	Germany	W1	266	98.52	4	1.48	270	7.20	0.007	0.11
		W2	260	94.20	16	5.80	276			
	Poland	W1	300	100.00	0	0.00	300	19.10	<0.001	0.17
		W2	320	93.84	21	6.16	341			
	Russia	W1	269	94.39	16	5.61	285	13.34	<0.001	0.15
		W2	233	85.04	41	14.96	273			
	Slovenia	W1	207	99.04	2	0.96	209	6.57	0.007	0.13
		W2	195	94.66	11	5.34	206			
	Turkey	W1	292	94.19	18	5.81	310	82.52	<0.001	0.36
		W2	202	64.74	110	35.26	312			
	Ukraine	W1	309	99.68	1	0.32	310	22.44	<0.001	0.19
		W2	306	92.17	26	7.83	332			
7. Job Loss Because of the Coronavirus										
	Total	W1	1157	68.71	527	31.29	1684	8.09	0.004	−0.05
		W2	1273	73.12	468	26.88	1741			
	Germany	W1	208	77.04	62	22.96	270	0.57	0.452	0.03
		W2	205	74.28	71	25.72	276			
	Poland	W1	217	72.33	83	27.67	300	0.00	0.977	0.00
		W2	247	72.43	94	27.57	341			
	Russia	W1	227	79.65	58	20.35	285	0.73	0.393	−0.04
		W2	226	82.48	48	17.52	273			
	Slovenia	W1	160	76.56	49	23.44	209	0.01	0.935	0.00
		W2	157	76.21	49	23.79	206			
	Turkey	W1	162	52.26	148	47.74	310	3.12	0.077	−0.07
		W2	185	59.29	127	40.71	312			
	Ukraine	W1	183	59.03	127	40.97	310	21.70	<0.001	−0.18
		W2	253	76.20	79	23.80	332			
8. Economic Status Worsened Due to the Pandemic										
	Total	W1	747	44.36	937	55.64	1684	0.02	0.885	0.00
		W2	768	44.11	973	55.89	1741			
	Germany	W1	202	74.81	68	25.19	270	1.19	0.275	0.05
		W2	195	70.65	81	29.35	276			
	Poland	W1	120	40.00	180	60.00	300	10.55	0.001	0.13
		W2	95	27.86	246	72.14	341			
	Russia	W1	124	43.51	161	56.49	285	11.78	<0.001	−0.15
		W2	159	58.03	115	41.97	273			
	Slovenia	W1	104	49.76	105	50.24	209	0.30	0.587	−0.03
		W2	108	52.43	98	47.57	206			
	Turkey	W1	109	35.16	201	64.84	310	0.08	0.783	−0.01
		W2	113	36.22	199	63.78	312			
	Ukraine	W1	88	28.39	222	71.61	310	0.10	0.752	−0.01
		W2	98	29.52	234	70.48	332			

**Table 3:** Descriptive statistics for coronavirus-related PTSD risk among university students in six countries during the second wave (October–December 2020) of the COVID-19 pandemic.

				95% CI					Cronbach’s
Sample	N	Range	M	LL	UL	SD	Skewness	Kurtosis	α
Total	1741	17–85	38.08	37.36	38.81	15.49	0.73	−0.29	0.94

Note. M = mean; CI = confidence interval; LL = lower limit; UL = upper limit; SD = standard deviation.

**Table 4:** Prevalence of coronavirus-related PTSD risk among university students in six countries during the second wave (October–December 2020) of the COVID-19 pandemic (*N* = 1741).

Cutoff Points for Coronavirus-Related PTSD Risk in the PCL-S	Frequencies of PTSD Risk among University Students from Various Countries			
	No PTSD Risk		PTSD Risk	
	*n*	%	*n*	%
Cutoff Point 25 (moderate risk)	380	21.80	1361	78.20
Cutoff Point 44 (high risk)	1171	67.30	570	32.70
Cutoff Point 50 (very high risk)	1339	76.90	402	23.10

**Table 5:** Results of logistic regression for coronavirus-related PTSD risk among university students during the second wave of the COVID-19 pandemic (W2) (*N* = 1741).

	Model 1: Moderate Risk					Model 2: High Risk					Model 3: Very High Risk				
	(PCL Cut−Off Point = 25)					(PCL Cut−Off Point = 44)					(PCL Cut−Off Point = 50)				
Variable	B	SE B	Χ^2^(1)	AOR	(95% CI)	B	SE B	Χ^2^(1)	AOR	(95% CI)	B	SE B	Χ^2^(1)	AOR	(95% CI)
Constant	−0.18	0.16	1.26	0.83		−2.12	0.18	135.88	0.12 ***		−2.51	0.21	148.56	0.08 ***	
Gender	0.66	0.15	19.35	1.94 ***	(1.44, 2.60)	0.69	0.15	20.54	2.00 ***	(1.48, 2.69)	0.57	0.17	11.35	1.78 ***	(1.27, 2.48)
Diagnosis of:															
Depression	0.14	0.29	0.23	1.15	(0.66, 2.01)	0.48	0.22	4.72	1.61*	(1.05, 2.47)	0.52	0.23	4.87	1.68*	(1.06, 2.65)
Anxiety	0.19	0.29	0.43	1.21	(0.68, 2.16)	−0.04	0.23	0.02	0.96	(0.62, 1.51)	−0.10	0.25	0.15	0.91	(0.56, 1.47)
PTSD	1.10	0.37	8.70	3.01 **	(1.45, 6.25)	0.37	0.24	2.31	1.45	(0.90, 2.33)	0.53	0.26	4.21	1.70 *	(1.02, 2.81)
Exposure to COVID−19															
1. Symptoms	0.49	0.17	7.92	1.63 **	(1.16, 2.29)	0.27	0.14	3.81	1.31	(1.00, 1.72)	0.23	0.15	2.23	1.25	(0.93, 1.69)
2. Testing	−0.40	0.20	4.04	0.67 *	(0.46, 0.99)	−0.13	0.17	0.59	0.87	(0.62, 1.23)	0.02	0.19	0.01	1.02	(0.70, 1.48)
3. Hospitalization	0.22	0.70	0.09	1.24	(0.31, 4.94)	−0.52	0.53	0.96	0.60	(0.21, 1.68)	−0.69	0.58	1.44	0.50	(0.16, 1.55)
4. Strict quarantine for at least 14 days	−0.38	0.25	2.33	0.68	(0.42, 1.12)	0.12	0.21	0.32	1.12	(0.75, 1.69)	0.03	0.23	0.02	1.03	(0.66, 1.61)
5. Friends or relatives infected	0.42	0.15	8.24	1.52 **	(1.14, 2.02)	0.00	0.13	0.00	1.00	(0.78, 1.29)	−0.14	0.14	0.92	0.87	(0.66, 1.15)
6. Death of friends or relatives	0.23	0.24	0.91	1.26	(0.79, 2.00)	0.53	0.17	9.54	1.71 **	(1.22, 2.39)	0.64	0.18	12.19	1.89 ***	(1.32, 2.70)
7. Job loss	0.32	0.18	3.36	1.38	(0.98, 1.95)	0.45	0.13	11.25	1.56 **	(1.20, 2.03)	0.60	0.14	17.52	1.82 ***	(1.37, 2.41)
8. Economic status worsened	0.93	0.14	43.46	2.52 ***	(1.92, 3.32)	0.65	0.13	24.93	1.91 ***	(1.48, 2.46)	0.59	0.15	15.96	1.80 ***	(1.35, 2.39)

* *p* < 0.05; ** *p* < 0.01; *** *p* < 0.001.

## Data Availability

The materials and methods are accessible at the Center for Open Science (OSF), titled: *Mental Health of Undergraduates During the COVID-19 Pandemic* [56]. The datasets generated during and/or analyzed during the current study are available from the corresponding author on reasonable request.

## References

[1] World Health Organization Rolling Updates on Coronavirus Disease (COVID-19). https://www.who.int/emergencies/diseases/novel-coronavirus-2019/events-as-they-happen.

[2] World Bank Global economic prospects, June 2020.

[3] Bonsaksen T., Leung J., Schoultz M., Thygesen H., Price D., Ruffolo M., Geirdal A.Ø. (2021). Cross-National Study of Worrying, Loneliness, and Mental Health during the COVID-19 Pandemic: A Comparison between Individuals with and without Infection in the Family. Healthcare.

[4] Hossain M.M., Tasnim S., Sultana A., Faizah F., Mazumder H., Zou L., McKyer E., Ahmed H.U., Ma P. (2020). Epidemiology of mental health problems in COVID-19: A review. F1000Research.

[5] Aristovnik A., Keržič D., Ravšelj D., Tomaževič N., Umek L. (2020). Impacts of the COVID-19 Pandemic on Life of Higher Education Students: A Global Perspective. Sustainability.

[6] Gloster A.T., Lamnisos D., Lubenko J., Presti G., Squatrito V., Constantinou M., Nicolaou C., Papacostas S., Aydın G., Chong Y.Y. (2020). Impact of COVID-19 pandemic on mental health: An international study. PLoS ONE.

[7] Adamson M.M., Phillips A., Seenivasan S., Martinez J., Grewal H., Kang X., Coetzee J., Luttenbacher I., Jester A., Harris O.A. (2020). International Prevalence and Correlates of Psychological Stress during the Global COVID-19 Pandemic. Int. J. Environ. Res. Public Health.

[8] Kavčič T., Avsec A., Kocjan G.Z. (2020). Psychological Functioning of Slovene Adults during the COVID-19 Pandemic: Does Resilience Matter?. Psychiatr. Q..

[9] International Labour Organization COVID-19 and the Education Sector. https://www.ilo.org/wcmsp5/groups/public/---ed_dialogue/---sector/documents/briefingnote/wcms_742025.pdf.

[10] Elani H.W., Allison P.J., Kumar R.A., Mancini L., Lambrou A., Bedos C. (2014). A systematic review of stress in dental students. J. Dent. Educ..

[11] Andrews B., Wilding J.M. (2004). The relation of depression and anxiety to life-stress and achievement in students. Br. J. Psychol..

[12] Borst J.M., Frings-Dresen M.H.W., Sluiter J.K. (2016). Prevalence and incidence of mental health problems among Dutch medical students and the study related and personal risk factors: A longitudinal study. Int. J. Adolesc. Med. Health..

[13] Vieira C.M., Franco O.H., Restrepo C.G., Abel T. (2020). COVID-19: The forgotten priorities of the pandemic. Maturitas.

[14] Fried E.I., Papanikolaou F., Epskamp S. (2021). Mental health and social contact during the COVID-19 pandemic: An ecological momentary assessment study. Clin. Psychol. Sci..

[15] Elmer T., Mepham K., Stadtfeld C. (2020). Students under lockdown: Comparisons of students’ social networks and mental health before and during the covid-19 crisis in Switzerland. PLoS ONE.

[16] Liang L., Ren H., Cao R., Hu Y., Qin Z., Li C., Mei S. (2020). The effect of COVID-19 on youth mental health. Psychiatr. Q..

[17] American Psychiatric Association (2000). Diagnostic and Statistical Manual of Mental Disorders (DSM-4^®^).

[18] American Psychiatric Association (2013). Diagnostic and Statistical Manual of Mental Disorders (DSM-5^®^).

[19] North C.S. (2016). Disaster mental health epidemiology: Methodological review and interpretation of research findings. Psychiatry Interpers. Biol. Process..

[20] North C.S., Surís A.M., Pollio D.E. (2021). A Nosological Exploration of PTSD and Trauma in Disaster Mental Health and Implications for the COVID-19 Pandemic. Behav. Sci..

[21] Shevlin M., Hyland P., Karatzias T. (2020). Is posttraumatic stress disorder meaningful in the context of the COVID-19 pandemic? A response to Van Overmeire’s commentary on Karatzias et al.. J. Trauma. Stress.

[22] Bridgland V., Moeck E.K., Green D.M., Swain T.L., Nayda D.M., Matson L.A., Hutchison N.P., Takarangi M. (2021). Why the COVID-19 pandemic is a traumatic stressor. PLoS ONE.

[23] Cooke J.E., Eirich R., Racine N., Madigan S. (2020). Prevalence of posttraumatic and general psychological stress during COVID-19: A rapid review and meta-analysis. Psychiatry Res..

[24] Chi X., Becker B., Yu Q., Willeit P., Jiao C., Huang L., Hossain M.M., Grabovac I., Yeung A., Lin J. (2020). Prevalence and Psychosocial Correlates of Mental Health Outcomes Among Chinese College Students During the Coronavirus Disease (COVID-19) Pandemic. Front. Psychiatry.

[25] Wathelet M., Fovet T., Jousset A., Duhem S., Habran E., Horn M., Debien C., Notredame C.E., Baubet T., Vaiva G. (2021). Prevalence of and factors associated with post-traumatic stress disorder among French university students 1 month after the COVID-19 lockdown. Transl. Psychiatry.

[26] Kimerling R., Ouimette P., Wolfe J. (2002). Gender and PTSD.

[27] Dell’Osso L., Carmassi C., Rucci P., Ciapparelli A., Paggini R., Ramacciotti C.E., Conversano C., Balestrieri M., Marazziti D. (2009). Lifetime subthreshold mania is related to suicidality in posttraumatic stress disorder. CNS Spectr..

[28] Dell’Osso L., Carmassi C., Massimetti G., Daneluzzo E., Di Tommaso S., Rossi A. (2011). Full and partial PTSD among young adult survivors 10 months after the L’Aquila 2009 earthquake: Gender differences. J. Affect Disord..

[29] DiGangi J.A., Gomez D., Mendoza L., Jason L.A., Keys C.B., Koenen K.C. (2013). Pretrauma risk factors for posttraumatic stress disorder: A systematic review of the literature. Clin. Psychol. Rev..

[30] Breslau N., Chilcoat H.D., Kessler R.C., Davis G.C. (1999). Previous exposure to trauma and PTSD effects of subsequent trauma: Results from the Detroit Area Survey of Trauma. Am. J. Psychiatry..

[31] Tang W., Hu T., Hu B., Jin C., Wang G., Xie C., Chen S., Xu J. (2020). Prevalence and correlates of PTSD and depressive symptoms one month after the outbreak of the COVID-19 epidemic in a sample of home-quarantined Chinese university students. J. Affect. Disord..

[32] Ochnik D., Rogowska A.M., Kuśnierz C., Jakubiak M., Schütz A., Held M.J., Arzenšek A., Benatov J., Berger R., Korchagina E.V. (2021). A Comparison of Depression and Anxiety among University Students in Nine Countries during the COVID-19 Pandemic. J. Clin. Med..

[33] World Values Survey.The Inglehart-Welzel World Cultural Map.World Values Survey 7. https://www.worldvaluessurvey.org/WVSNewsShow.jsp?ID=428.

[34] Hale T., Petherick A., Phillips T., Webster S. (2020). Variation in government responses to COVID-19. Blavatnik Sch. Gov. Work. Pap..

[35] Dong E., Du H., Gardner L. (2020). An interactive web-based dashboard to track COVID-19 in real time. Lancet Infect. Dis..

[36] Faul F., Erdfelder E., Lang A.G., Buchner A. (2007). G*Power 3: A flexible statistical power analysis program for the social, behavioral, and biomedical sciences. Behav. Res. Methods.

[37] Rogowska A.M., Pavlova I., Kuśnierz C., Ochnik D., Bodnar I., Petrytsa P. (2020). Does Physical Activity Matter for the Mental Health of University Students during the COVID-19 pandemic?. J. Clin. Med..

[38] Beaton D.E., Bombardier C., Guillemin F., Ferraz M.B. (2000). Guidelines for the process of cross-cultural adaptation of self-report measures. Spine.

[39] Weathers F.W., Ruscio A.M., Keane T.M. (1999). Psychometric properties of nine scoring rules for the Clinician-Administered Posttraumatic Stress Disorder Scale. Psychol. Assess..

[40] Prins A., Kimerling R., Yeager D.E., Magruder K.M. (2010). Guidelines for Interpreting PCL Scores in VA Settings: An Interval Approach.

[41] Blanchard E.B., Jones-Alexander J., Buckley T.C., Forneris C.A. (1996). Psychometric properties of the PTSD checklist (PCL). Behav. Res. Ther..

[42] Fritz C.O., Morris P.E., Richler J.J. (2012). Effect size estimates: Current use, calculations, and interpretation. J. Exp. Psychol. Gen..

[43] Aslan I., Ochnik D., Çınar O. (2020). Exploring Perceived Stress among Students in Turkey during the COVID-19 Pandemic. Int. J. Environ. Res. Public Health.

[44] The New York Times. https://www.nytimes.com/2020/04/20/world/middleeast/coronavirus-turkey-deaths.html.

[45] The Economist. https://www.economist.com/graphic-detail/2020/07/15/tracking-covid-19-excess-deaths-across-countrie.

[46] Liu C., Zhang E., Wong G., Hyun S., Hahm H. (2020). Factors associated with depression, anxiety, and PTSD symptomatology during the COVID-19 pandemic: Clinical implications for U.S. young adult mental health. Psychiatry Res..

[47] Liu N., Zhang F., Wei C., Jia Y., Shang Z., Sun L., Wu L., Sun Z., Zhou Y., Wang Y. (2020). Prevalence and predictors of PTSS during COVID-19 outbreak in China hardest-hit areas: Gender differences matter. Psychiatry Res..

[48] Forte G., Favieri F., Tambelli R., Casagrande M. (2020). COVID-19 pandemic in the Italian population: Validation of a post-traumatic stress disorder questionnaire and prevalence of PTSD symptomatology. Int. J. Environ. Res. Public Health.

[49] Li Q. (2020). Psychosocial and coping responses towards 2019 coronavirus diseases (COVID-19): A cross-sectional study within the Chinese general population. QJM.

[50] Zhang L., Pan R., Cai Y., Pan J. (2021). The Prevalence of Post-Traumatic Stress Disorder in the General Population during the COVID-19 Pandemic: A Systematic Review and Single-Arm Meta-Analysis. Psychiatry Investig..

[51] GUS Higher Education and its finances in 2019. https://stat.gov.pl/obszary-tematyczne/edukacja/.

[52] Statista Ratio of women in Germany in 2015 according to academic track gender. https://de.statista.com/.

[53] SURS Student Enrolment in Tertiary Education, Slovenia, Academic Year 2020/2021 Republic of Slovenia Statistical Office. https://www.stat.si/StatWeb/en/News/Index/9537.

[54] The Knesset Research and Information Centre Representation of Women in the Israeli Academia. https://m.knesset.gov.il/EN/activity/mmm/me040618.pdf.

[55] Szmyd B., Bartoszek A., Karuga F.F., Staniecka K., Błaszczyk M., Radek M. (2021). Medical Students and SARS-CoV-2 Vaccination: Attitude and Behaviors. Vaccines.

[56] Ochnik D., Rogowska A.M., Kuśnierz C., Jakubiak M., Pavlova I., Arzenšek A., Blažková I., Korchagina E., Schütz A., Aslan I. (2020). Mental health of Undergraduates during the COVID-19 Pandemic.

